# Analysis of sales of antimicrobials used in veterinary medicine in Poland compared to Europe

**DOI:** 10.2478/jvetres-2026-0032

**Published:** 2026-06-30

**Authors:** Ilona Barbara Materek, Katarzyna Pasik, Ewa Borzym

**Affiliations:** Department of Veterinary Medicinal Products Control, National Veterinary Research Institute, 24-100 Puławy, Poland

**Keywords:** AMR, ESVAC/ESUAvet, One Health, veterinary antimicrobials

## Abstract

**Introduction:**

Antimicrobial resistance (AMR) poses a global threat to public and animal health, and its progression is closely linked to the use of antimicrobials in veterinary medicine. In Europe, programmes such as the European Surveillance of Veterinary Antimicrobial Consumption (ESVAC) and subsequently European Sales and Use of Antimicrobials for Veterinary Medicine (ESUAvet) monitor and attempt to reduce the consumption of antimicrobials in food-producing animals. The study analyses the trends in the volume and sales structure of veterinary antimicrobials in Poland in 2011–2023 and compares them with data reported by other European countries.

**Material and Methods:**

The analysis was based on data from ESVAC reports (2011–2022) and the first ESUAvet report (2023), as well as data from the European Medicines Agency’s Antimicrobial Sales and Use Platform. The data were evaluated by antimicrobial class, pharmaceutical form and Antimicrobial Advice Ad-hoc Expert Group (AMEG) categories, the latter being A (avoid), B (restrict), C (caution) and D (prudence).

**Results:**

While total sales of veterinary antimicrobials in Europe decreased by 48.0% between 2011 and 2023, in Poland they increased by 19.5% during the same period. In Poland, the predominance of AMEG category D antimicrobials persisted, although their share declined from 79% to 64% with a concurrent increase in sales of category C. The sales structure shifted significantly towards oral solutions, replacing oral powders. Penicillins became the dominant antimicrobial group in the later years, while tetracyclines sales declined.

**Conclusion:**

The Polish trend diverges from the European one of reducing veterinary antimicrobial consumption. To align with EU reduction targets, Poland should strengthen antimicrobial stewardship, tighten prescription control and promote preventive measures such as vaccination and higher biosecurity within the One Health framework.

## Introduction

The discovery of antibiotics nearly 100 years ago has saved many lives around the world (30). In parallel with the development of vaccinations, antibiotics have contributed to increasing life expectancy by almost 23 years (27). However, coinciding with the introduction of the first modern antimicrobials, the process of treating infectious diseases has been hampered by antimicrobial resistance (AMR), including antibiotic resistance (2, 26). It has been found that bacteria use both phenotypic and genetic mechanisms to develop natural defences against applied antibiotics, and thereby raise their resistance to these agents (40). The widespread and inappropriate use of antimicrobials in humans and food-producing animals is a major factor contributing to AMR. The scale of the consequences of improper use is enormous. Antimicrobial resistance causes over 35,000 deaths in Europe and 10 million deaths worldwide annually (29, 36). Furthermore, the impact of AMR on public health is comparable to that of HIV/AIDS, influenza and tuberculosis combined (37). Addressing AMR requires a coordinated effort from the human and animal health sectors worldwide (4). It was necessary to establish both European Union (EU) legal provisions in veterinary legislation and relevant guidelines to safeguard the effectiveness and availability of antibiotics (36).

The European Medicines Agency (EMA) plays a vital role in the global response to the spread of AMR, supporting the One Health approach and promoting close cooperation between the veterinary and human sectors (29, 37). In 2009, the EMA launched the European Surveillance of Veterinary Antimicrobial Consumption (ESVAC) project. The goal of this project was to compile annual reports on sales of veterinary antimicrobials from 31 countries (38). Data from the ESVAC project showed the volume of sales of veterinary antimicrobials in EU member states and European Economic Area (EEA) countries. This information was considered essential to identify possible risk factors that could lead to the development and spread of AMR in animals (36). As part of the coordinated EU response to AMR, the European Commission has set targets as part of its actions to combat the problem. One is the aspirational target of a 50% reduction in overall EU sales of antimicrobials for use in livestock farming and in aquaculture by 2030, with 2018 as the reference year and 118.3 mg/population correction unit (PCU) as the reference value (10).

As a result of work on reducing the use and controlling the distribution of antimicrobials in veterinary medicine, in 2014 the Antimicrobial Advice Ad Hoc Expert Group (AMEG) categorised antimicrobial active substances into four tiers (A, B, C and D) according to their permissible use (9). Category A (avoid) includes substances that cannot be used in food-producing animals and should only be administered to individual companion animals in exceptional cases. For this reason, neither in the ESVAC project nor later, were data on volume of sales of antimicrobials belonging to category A collected. Category B (restrict) includes substances which are critically important in human medicine and their use in animals should be limited to reduce the risk to public health. They may be used when antimicrobials in categories C and D are ineffective or when a given pathogen is sensitive only to an antimicrobial from this class. Most substances listed in this group are classified as highest-priority and critically important. These include polymyxins, quinolones and third- and fourth-generation cephalosporins excluding combinations with beta-lactamase inhibitors being graded with this description. Category C (caution) antimicrobials should be used only when no antimicrobial substances from category D would be clinically effective. The final group - category D (prudence), consists of antimicrobial substances of which the use is associated with a low risk of reduced susceptibility in pathogenic bacteria. They should preferably be used as first-line treatments whenever possible. The category is characterised by a high safety profile, but they should regardless not be used unnecessarily (9, 25).

A milestone in countering AMR was the adoption of the new veterinary Regulation (EU) 2019/6 of the European Parliament and of the Council of 11 December 2018 on veterinary medicinal products and repealing Directive 2001/82/EC (22). It promotes reducing or completely eliminating the use of antimicrobials, including their use as feed additives for farm animals, as well as in prophylaxis, metaphylaxis and treatment (4). Regulation (EU) 2019/6, commonly known as the New Veterinary Regulation, was signed on 11 December 2018 and entered into force on 28 January 2022. It concerns the issuing of marketing authorisations and the monitoring of the sales and use of veterinary medicinal products throughout the EU (Table 1) (38). Article 57 of Regulation (EU) 2019/6 requires member states to collect and report data on the volume of sales and on the use of antimicrobial medicinal products used in animals. Previously, data were reported voluntarily under the ESVAC project. The data are provided to the EMA to enable the real assessment of the consumption of such products in food-producing animals. The new program of annual surveillance reports with mandatory reporting of antimicrobial sales and use is the ESUAvet initiative. The legal provisions referred to apply to all the 27 EU countries, Iceland and Norway (10, 37). The aim of this study was to analyse the trends in the volume and structure of sales of antimicrobial veterinary medicinal products (VMPs) in Poland in the years 2011–2023 in comparison with data reported by other European countries.

**Table 1.** Main obligations of EU member states to reduce antimicrobial resistance imposed by Regulation (UE) 2019/6 (22)

1.	To only allow preventive use of antimicrobials in single animals and small groups following veterinary assessment
2.	To restrict the metaphylactic use of antimicrobials
3.	To ban specific antimicrobials from non-humans use (electively)
4.	To collect data on the sales and use of antimicrobials
5.	To prohibit the import of animals and animal products from third countries where antimicrobials have been used in a manner not compliant with EU rules

## Material and Methods

This article analyses data on the volume of sales of veterinary antimicrobials from 2011 to 2022 extracted from the ESVAC reports published annually by the EMA (11-21). Data for 2023 were sourced from the first ESUAvet report following changes in the legal framework (10) and from the Antimicrobial Sales and Use Platform, a new informatic tool developed by EMA to support the mandatory collection and reporting of data on antimicrobial products in animals from the EU/EEA (7, 8). These data help strengthen EU action against AMR in humans and animals under the One Health approach (42). Data from the 2011–2022 period were collected uniformly for all countries participating in the ESVAC project and provide the most comprehensive available evidence on pan-European volume of sales of veterinary antimicrobials. The sales trends for Poland were analysed in the context of this European data.

The current legal framework for collecting and publishing data on the volume of sales and use of veterinary antimicrobials can be found in three main legal acts: Article 57 of Regulation (EU) 2019/6 of the European Parliament and of the Council of 11 December 2018 on veterinary medicinal products and repealing Directive 2001/82/EC (22), Commission Delegated Regulation (EU) 2021/578 of 29 January 2021 supplementing Regulation (EU) 2019/6 of the European Parliament and of the Council with regard to requirements for the collection of data on the volume of sales and on the use of antimicrobial medicinal products in animals (5) and Commission Implementing Regulation (EU) 2022/209 of 16 February 2022 establishing the format of the data to be collected and reported in order to determine the volume of sales and the use of antimicrobial medicinal products in animals in accordance with Regulation (EU) 2019/6 of the European Parliament and of the Council (6). Pursuant to the Regulation of the Polish Minister of Agriculture and Rural Development of 16 December 2016 (24), information on the volume of sales of VMPs is submitted quarterly by entities trading wholesale in these products in the form of electronic reports sent to the National Veterinary Research Institute in Puławy. As of January 2026, there are 96 pharmaceutical wholesalers of VMPs operating in the Republic of Poland (23). However, across Europe, more diverse sources than only wholesalers report VMPs sales data (Table 2) (10).

**Table 2.** Providers of the data on the volume of sales of veterinary antimicrobials reported by EU member states and EEA countries to the European Medicines Agency for 2023 (10, 11)

Data provider	Countries
Marketing Authorisation Holders	Austria, Belgium, Bulgaria, Croatia, France, Germany, Greece, Ireland, Netherlands, Portugal, Romania, Spain
Wholesalers	Austria, Bulgaria, Croatia, Cyprus, Czechia, Estonia, Finland, Germany, Greece, Hungary, Iceland, Latvia, Lithuania, Luxembourg, Malta, Norway, Poland, Portugal, Slovakia, Slovenia
Feed mills	Belgium, Cyprus, Czechia, Italy, Malta, Norway
Pharmacies	Denmark, Italy, Sweden
Retailers	Italy
Veterinarians	Sweden

Reports on the volume of sales of VMPs submitted by member states and EEA countries to the EMA included the marketing authorisation number, international trade name, dose and dosage form, active substances and their strengths. Sales were recorded by anatomical therapeutic chemical (ATC) classification codes (33) and expressed as the numbers of packages sold (8, 22). Total EU and EEA sales in 2023 of those VMPs mandatory to report include three additional ATCvet codes (QA07AX03, QA07AX04 and QJ54) of veterinary antimicrobials (Table 3) (10, 11). The VMPs for which the collection of volume of sales data and their reporting to the EMA is now mandatory or voluntary are specified in Annexes 3 and 4 of the Commission Delegated Regulation (EU) 2021/578, respectively (5). The mandatorily reported VMPs are antibacterials, antiprotozoals with antibacterial effect, intramammary antimycobacterials and some anti-infective agents, all of which have bacteriostatic or bactericidal activity. The voluntarily reported VMPs are antivirals, antifungals, topical antibacterials, antiprotozoals and the remaining anti-infectives (Table 3). In 2023, all member states and two EEA countries reported sales data for VMPs mandatory to report. Additionally, 17 countries also reported data for the products voluntary to report. Poland reported only the sales volume VMPs which it was obliged to.

**Table 3.** Antimicrobial veterinary medical products based on the ATCvet classification (5) grouped as products mandatory to report sales of and products voluntary to report sales of (5)

Reporting	Antimicrobial substance group	ATCvet code
	antidiarrhoeals, intestinal anti-inflammatory and anti-infective agents	QA07AA, QA07AB, QA07AX03, QA07AX04
	gynaecological anti-infectives and antiseptics	QG01AA, QG01AE, QG01BA, QG01BE
	anti-infectives and antiseptics for intrauterine use	QG51AA, QG51AG
Mandatory	antibacterials for systemic use	QJ01
	antibacterials for intramammary use	QJ51
	antiprotozoals (with antibacterial effect)	QP51AG
	antimycobacterials for intramammary use	QJ54
	antiprotozoals (other than QP51AG)	QP51
	antifungals for topical use	QD01A
	antifungals for systemic use	QD01B
	antimycotics for systemic use	QJ02
	antimycobacterials	QJ04
Voluntary	antivirals for systemic use	QJ05
	antibiotics and chemotherapeutics for dermatological use	QD06
	other nasal preparations	QR01AX06, QR01AX08
	ophthalmological anti-infectives	QS01AA, QS01AB, QS01AD, QS01AE, QS01CA, QS01C
	otological anti-infectives	QS02AA, QS02CA, QS03AA, QS03CA

In 2023, the EMA published a guideline revising the method for calculating animal biomass denominators for presenting population-adjusted sales and use data in the ESUAvet annual reports. The method changes the denominator, replacing the PCU (a standardised estimate of animal population weight based on fixed species-specific assumptions) with animal biomass (an estimate of population weight including additional species and categories and updated weights). As a result, the sales indicator in the ESUAvet reports is mg/kg and not directly comparable to the mg/PCU indicators in the previous ESVAC reports. The ESUAvet denominator aligns more closely with the denominator used by the World Organisation for Animal Health (WOAH) in reporting global data on antimicrobials intended for use in animals (10). Such methodological differences impair the direct comparability of indicators over time, especially when analysing long-term trends that span both reporting frameworks. However, in the first ESUAvet report, the volume of sales of veterinary antimicrobials was presented both in mg/PCU and mg/kg (10). Antimicrobial sales in mg/PCU were analysed according to ATC class and subclass, product form and EMA classification system (33).

All statistical analyses and calculations were conducted using Microsoft Excel 2019 software. The results are presented as tables and graphs.

## Results

**Fig. 1.**
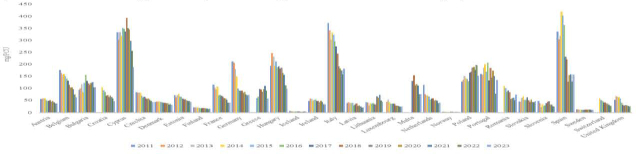
Overall sales of antimicrobial veterinary medical products primarily for food-producing animals for EU and EEA countries reported in mg/PCU during 2011–2023 (10–21). PCU - population correction unit

During the period 2011–2023, there was a marked decline in total antimicrobial sales in European countries (10). In the 25 countries that provided data continuously, a general downward trend was observed (Fig. 1). Total sales of VMPs decreased by approximately 48%, from 161.2 mg/PCU in 2011 to 84.6 mg/PCU in 2023. Twelve countries showed a decrease of more than 40% (Table 4) compared to their sales data reported for 2011. Throughout this period, sales increased only in Bulgaria (from 92.6 mg/PCU in 2011 to 103.6 mg/PCU in 2023, making an 11.9% increase) and in Poland (from 126.3 mg/PCU in 2011 to 150.9 mg/PCU in 2023, representing a 19.5%). The upward trend in sales of VMPs for Poland could be particularly clearly observed between 2017 and 2022. The volume of sales reported by Poland in 2023 was approximately two-fold higher than the European mean (150.9 mg/PCU *versus* 84.6 mg/PCU reported for the EU, Iceland and Norway). Based on data from the ESVAC reports, all six countries (Croatia, Greece, Luxembourg, Malta, Romania and Switzerland) that joined ESVAC after 2011 also recorded a decrease in the volume of sales of antimicrobials compared with their initial reporting years (10, 15, 17, 18, 20).

**Table 4.** Decrease in antimicrobial veterinary medicinal product sales in EU and EEA countries between 2011 and 2023 (10, 21)

0–20.0%	20.1–40.0%	40.1–60.0%	60.1–80.0%
Portugal (16.2%)	Austria (33.8%)	Czechia (49.2%)	Belgium (64.7%)
Sweden (14.5%)	Denmark (24.2%)	Cyprus (43.8%)	Germany (66.4%)
	Estonia (39.7%)	Hungary (49.6%)	France (70.4%)
	Finland (24.4%)	Italy (51.4%)	Netherlands (65.3%)
	Iceland (28.3%)	Latvia (41.4%)	
	Ireland (28.0%)	Slovenia (47.2%)	
	Norway (40.0%)	Spain (53.4%)	
		United Kingdom (49.6%)^*^	

* – until 2022

A comparison of selected EU countries shows that the increase in sales in Poland in the years 2011–2023 contrasts clearly with the consistent decline observed in as the country’s western neighbours: Germany, France and Belgium (Table 5).

**Table 5.** Comparison of antimicrobial veterinary medicinal product sales indicators in Poland and selected EU countries (11)

Country	2011 sales (mg/PCU)	2023 sales (mg/PCU)	2011–2023 trend
Poland	126.3	150.9	Increasing
Belgium	175.1	61.8	Decreasing
Denmark	42.1	31.9	Decreasing
Germany	211.5	71.1	Decreasing
France	133.6	39.5	Decreasing

1 PCU – population correction unit

The volume of sales of VMPs varied substantially over the analysed period in both Poland and Europe (using a European mean to be representative) (Table 6). In Poland, annual changes ranged from marked increases, such as in 2013 (+14.4%) and 2017 (+27.6%), to notable decreases, including 2014 (−7.7%), 2016 (−6.9%) and 2023 (−23.0%). Rises in sales predominated, particularly between 2017 and 2022. In contrast, the European mean fell more frequently than rose year to year, with pronounced reductions observed in 2012 (−19.3%), 2014 (−17.6%), 2017 (−14.1%) and 2019 (−18.4%). Although some years saw increases at the European level, these were generally followed by decreases. Overall, the year-to-year dynamics illustrate divergent trajectories, with Poland showing greater variability and a predominance of increases, and the European mean being characterised by a more consistent downward pattern over the analysed period.

**Table 6.** Year-to-year percentage changes (%) in antimicrobial veterinary medicinal product sales (mg/PCU) in Poland and in Europe during 2011–2023 (10–21)

Region	2011	2012	2013	2014	2015	2016	2017	2018	2019	2020	2021	2022	2023
Poland	123.6	+7.0	+14.4	−7.7	−1.1	−6.9	+27.6	+2.7	+10.0	+1.5	−6.6	+11.7	−23.0
Rest of EU + EEA(mean)	161.2	−19.3	+0.6	−17.6	+28.8	−10.3	−14.1	−3.6	−18.4	+5.7	−5.2	−12.4	+14.5

**Fig. 2.**
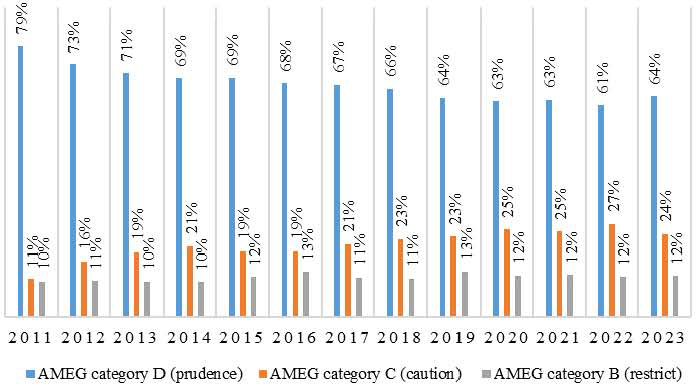
Sales of antimicrobial veterinary medical products in Poland during 2011–2023 excluding preparations in AMEG category A (avoid) (10–21)

From 2011 to 2023, sales of VMPs in Poland were dominated by products classified in category D, of which the proportion gradually declined from approximately 79% in 2011 to 64% in 2023. Despite this decline, products in this category still constituted the vast majority of the market. Conversely, category C showed an increasing trend, from 11% in 2011 to 24% in 2023. Category B, comprising substances of the highest importance to human medicine, accounted for a small, stable proportion throughout the analysed period, of approximately 10–12% (Fig. 2). No major changes in trend were observed in this category. There was a gradual change in Poland in the sales structure towards greater sales of category C products and commensurately lower sales of category D ones. A low sales volume of category B VMPs was constant.

The EMA classification of pharmaceutical forms takes into account both a drug’s form and its route of administration. Oral solutions include liquid preparations, powders and concentrates intended for dissolution in drinking water, milk or milk replacer, and are primarily used in group therapy. Oral powders are preparations administered exclusively with feed. This classification allows differentiation between drug administration methods and better interpretation of sales trends in individual animal production sectors (8, 10).

**Fig. 3.**
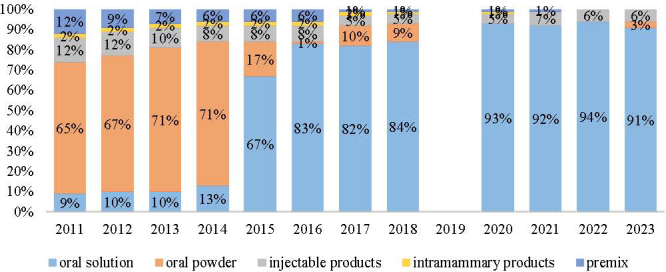
Proportions of Polish sales of antimicrobial veterinary medicinal products primarily for food-producing animals by administration route during 2011–2023 (10–21). Data for 2019 were not available

In the early years of the analysed period (2011–2014), oral powders dominated, accounting for 65% to 71% of total sales of VMPs in Poland. At the same time, oral solutions (approximately 9–13%) and injectable products (10–12%) were relatively small proportions of the volume of sales. The proportions of other categories, such as intramammary products and premixes, was marginal (1–2%). From 2015, a shift in the sales structure was observed. The sales of oral solutions increased to 67% in that year, and remained very high in subsequent years (82–94%), peaking in 2020–2022. At the same time, there was a marked decline in sales of oral powders, which accounted for less than 10% after 2015. Sales of injectable products gradually declined, from approximately 12% in 2011 to 6–7% in recent years. Intramammary preparations maintained their small proportion (1–3%) throughout the period (Fig. 3).

**Fig. 4.**
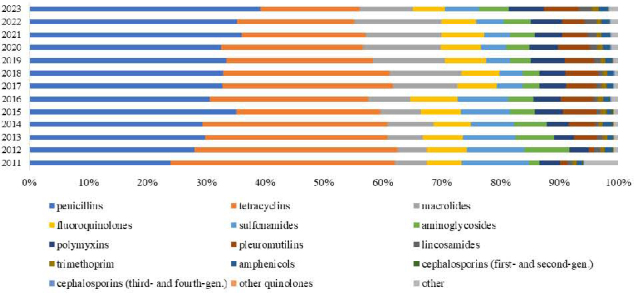
Proportions of sales of antimicrobial veterinary medicinal products primarily for food-producing animals by antimicrobial class in Poland during 2011–2023 (10–21)

In the first three years of the analysed period, tetracyclines were the most sold, accounting for more than 30–40% of total sales. Penicillins were another significant slice of antibiotic sales (approximately 25–30%), while sulphonamides, macrolides, fluoroquinolones and aminoglycosides had significantly smaller volumes. During this period, polymyxins and first- and second-generation cephalosporins were also sold, but in yet lower volumes. From around 2015, a clear shift in sales structure was observed. The proportion which was tetracyclines began to gradually shrink, reaching 17% in 2023, while the proportion which was penicillins enlarged significantly, accounting for almost half of total VMPs sales in Poland in 2023. Macrolides were sold as a relatively stable proportion (approximately 10–15%) throughout most of the analysed period, while fluoroquinolones and third- and fourth-generation cephalosporins, considered critically important agents for human medicine, had a low share of the market (approximately 5%). Small but consistent market shares were also noted for pleuromutilins and polymyxins, which increased over the analysed period. Lincosamides, amphenicols and trimethoprim comprised marginal, relatively stable, proportions of sales (Fig. 4).

## Discussion

Between 2011 and 2023, total sales of VMPs in Europe trended clearly downward, reflecting the effectiveness of EU-wide initiatives aimed at reducing their use in food-producing animals (10). To date, approximately half of the 50% reduction target for 2030 has already been achieved. Many western and northern European countries, including Denmark, Sweden and Norway, have maintained consistently low consumption levels, often below the European mean. This is the result of long-standing regulatory frameworks, strict prescription control and the implementation of national reduction programmes (3, 34, 39). Similarly, several central and western European countries achieved gradual reductions over the study period, confirming the success of EU-coordinated policy interventions.

Poland’s trajectory diverged from that of Europe overall. While the European mean sales volume steadily decreased, Poland’s volume persistently increased between 2011 and 2023. This suggests that national actions had not yet achieved the desired reduction in VMPs sales. Poland consistently remained above the European mean, ranking among the highest-consuming countries in the EU and EEA. The growing sales in Poland likely reflected a combination of factors, including the influence of the country’s geographical location. As a border country of the EU, Poland witnesses the transit of animals and animal products between the eastern and western parts of the continent. High animal mobility and intensive export-oriented production can increase the epidemiological pressure and drive the introduction of pathogens, which may lead to the use of group therapies in large herds. The impact of Poland’s geographical location on the volume of sales of VMPs is indirect and interacts with other factors, such as the structure of animal production, biomass size and national herd health management practices.

When interpreting the extent and dynamics of veterinary antimicrobials sales, the impact of national animal biomass and the structure of animal production should be considered. Poland has a distinct agricultural sector structure, with intensive poultry production playing a particularly important role. Poland is the largest poultry producer in the EU. Polish production accounts for approximately 20–21% of total EU poultry production and has an annual output of around 2.7–2.8 million tonnes of poultry meat, significantly more than other major producers such as Spain, Germany or France (40). This large share of poultry production, coupled with significant export volumes, highlights the dominant role of the poultry sector in Polish livestock agriculture and its potential influence on aggregated antimicrobial sales. The lack of data enabling the attribution of sales of individual products to intended use in poultry, turkeys, cattle or pigs specifically is a significant limitation of the surveillance system based on sales data, which prevents direct assessment of sales at the sectoral level.

The observed disparities between Poland and low-use countries underline the importance of strengthening national antimicrobial reduction strategies. Data from Scandinavian and some western European countries demonstrate that significant reductions can be achieved without compromising animal health, productivity or biosecurity, provided that vaccination and monitoring systems are effectively implemented (32).

Considering VMPs sales AMEG category by category, the largest sales were of category D products both in Poland and the rest of Europe and the EEA. This category includes substances considered suitable for routine veterinary use. In Europe, preparations in this category comprised a relatively stable 67–72% of all sales between 2016 and 2023. In Poland, although it remained the dominant category until the period end, a clear downward trend was observed, from 79% in 2011 to 64% in 2023. This indicates that the Polish VMPs market is diversifying towards products in more restrictive categories more quickly than those of other European countries. In Poland and outside it, there was a noticeable and commensurate increase in the size of the market for VMPs in category C, which includes substances requiring careful handling and restricted use. In Europe, this category accounted for approximately 19–25% of sales during the analysed period, and this proportion remained rose relatively stable over the 12 years. In Poland, however, the share of category C drugs increased by significantly more, from 11% in 2011 to 24–27% in recent years. The burgeoning market for category C veterinary antimicrobials may be interpreted in several ways. While this trend could be consistent with more selective therapeutic choices in situations where first-line agents are no longer suitable, alternative explanations should also be considered. Changes in market availability, including the introduction of new VMPs or the withdrawal of older formulations, may influence sales patterns independently of choice exercised by the prescriber. In addition, pharmaceutical marketing strategies and shifts in product promotion could affect the relative use of specific antimicrobial classes. Given the aggregated and sales-based nature of the data, it is not possible to disentangle these factors. Therefore, interpretations regarding therapeutic precision should be regarded as cautious and hypothetical (10–21). Category B, which includes substances of the highest importance to human medicine, comprises drugs taking a low, stable, approximate 6–7% market share in Europe and 10–12% in Poland. The higher share of this category in Poland may be due to differences in the structure of animal production (large extent of intensive poultry and pig farming) and the availability of specific therapeutic agents (11).

The results of the analysis of veterinary antimicrobial sales indicate significant changes in veterinary medicine prescribing practice in Poland over the last decade. The dominance of oral solutions observed between 2015 and 2023 reflects their widespread use in intensive animal production systems, particularly in the poultry and pig sectors, where group therapy administered *via* drinking water is favoured. This method of administration allows rapid treatment of large herds, but it also carries the risk of less precise dosing compared to individual therapy (10–21). The larger proportion of sales of this pharmaceutical form, coupled with a simultaneous contraction in sales of oral powders, may indicate the implementation of principles of rational antimicrobial use and reduction of the risks associated with uncontrolled mixing of drugs in feed or water. This transformation is consistent with the European Union’s policy directions for combating antimicrobial resistance (22, 41). The decline in the market share of injectable products observed since 2015 can be interpreted in two different ways. On the one hand, it may indicate the use of individual therapies, which helps reduce the exposure of staff and animals to antibiotics critical to human medicine. On the other hand, it may be a sign of less precise treatment of infections requiring targeted drug administration. The persistently low percentage of intramammary products indicates that their profile in the overall use of VMPs remained low. This may be due to the concentration of preventive measures on dairy farms and the introduction of biosecurity programmes, both of which reduce the need for clinical treatment of mastitis (43).

In Poland, sales of penicillins and tetracyclines made up over half of all sales between 2011 and 2023 (10–21). In the early years of the analysed period, the volume of tetracyclines was particularly large, but over time, a gradual rise in the volume of penicillins was observed. At the same time, there was a noticeable decline in sales of sulphonamides, as well as a reduction in the use of fluoroquinolones and third- and fourth-generation cephalosporins, classified as category B (restricted). This phenomenon demonstrates the effectiveness of the measures implemented in Poland to rationalise antimicrobial use in veterinary medicine, including the recommendations of the national programme for monitoring consumption and selection pressure, as well as EU regulations regarding the use of critically important antibiotics. In Europe, the overall structure of antimicrobial sales in veterinary medicine was similar, although the proportions of individual groups of substances fluctuated less. From 2016 to 2023, sales of tetracyclines (approximately 30–40%) and penicillins (approximately 30%) dominated, trailed by sales of a significant but stable share of macrolides. During the period, European countries observed a steady decline in sales of substances with a high potential to select for resistance, particularly polymyxins, third- and fourth-generation cephalosporins and fluoroquinolones (10–16, 42). Compared to Europe, Poland demonstrated more dynamic changes in its sales structure, with a particularly noticeable increase in the purchase of penicillins and parallel decrease in use of tetracyclines, as well as a marked reduction in the buying of category B VMPs. Both in Poland and Europe, the market share of broad-spectrum antimicrobials (especially penicillins, tetracyclines and macrolides), which can generate selection pressure when used against zoonotic and environmental pathogens, remained high.

The entry into force of Regulation (EU) 2019/6, alongside national measures aimed at prudent antimicrobial use, may have contributed to the continued decline in VMPs sales in several member states. Since the adoption of Regulation (EU) 2019/6 on veterinary medicinal products in 2018, the largest declines in veterinary antimicrobials sales between 2019 and 2023 were observed in Hungary (47.5%), Cyprus (46.7%), and Slovenia (45.9%). Data reported for Poland indicated an 18.5% decrease in the volume of sales of veterinary antimicrobials in 2023 compared to 2019. The constancy of sales with no reduction seen between 2019 and 2023 in Spain or Romania (10, 18, 21) may reflect an insignificant impact of new EU regulations on national policies.

Partial data on the volume of sales of VMPs in Poland have been analysed previously. An analysis of their sales in 2021 showed that Poland was one of four countries where a volume of sales of VMPs increase compared to previous years was observed (12). Hypothetically, it may reflect a combination of post-pandemic intensification of livestock production, which could have led to increased disease pressure in large herds, especially in the poultry and pig sectors, increased use of group treatments and the market and regulatory factors before the full implementation of the new regulations resulting from Regulation (EU) 2019/6. In 2021, the high sales rate of third-generation cephalosporins was particularly concerning. Although the indicator for third-generation cephalosporins was relatively small (the mean for 31 countries in 2020 was 0.2 mg/PCU), in Poland it was twice as high at 0.4 mg/PCU, and represented a fourfold increase over the 2011 indicator. Fluoroquinolone sales also saw a dynamic increase. Poland saw the largest increase in sales of this group of antimicrobials. A similar comparison of the quantities of polymyxins sold showed a significant upward trend, especially in Poland (the indicator rising from 4.1 to 9.1 mg/PCU), Cyprus (from 8.2 to 15.9 mg/PCU), Bulgaria (from 3.2 to 5.4 mg/PCU) and Portugal (from 7.9 to 11.7 mg/PCU) (35). Analysis of data for 2022 showed a significant increase in VMPs sales in Poland in just one year (from 2021 to 2022), reaching 11.7% (from 175.5 mg/PCU to 196.0 mg/PCU). A continuing concern was the unabating high levels of Polish consumption of fluoroquinolones, macrolides, penicillins and polymyxins. The only antimicrobial group to be sold less were quinolones (25). The observed fluctuations may be partially due to differences in distribution channels or purchasing behaviour, rather than solely to changes in antimicrobial use practices.

Of particular interest are the strategies of Germany, France and Belgium aimed at limiting the sales of antimicrobials. In all three countries, the volume of sales of veterinary antimicrobials in 2011 was higher than it was in Poland (21). These countries systematically and significantly reduced their sales volume by 2023, by 64.7%, 66.4% and 70.4%, respectively. An example of systematic commitment is that in Germany, a national system for monitoring the volume of sales of antimicrobials was established in 2011. In addition, two legally binding measures were introduced to control the risk of antimicrobial resistance resulting from the use of antimicrobials in veterinary medicine: the German benchmarking of treatment frequency in 2014 and the obligation to perform susceptibility testing of bacteria to third- and fourth-generation cephalosporins and fluoroquinolones in 2018. In 2022, Germany implemented the Veterinary Medicinal Products Act (28). France successfully reduced sales of antimicrobials through national plans like Écoantibio, achieving a 52% decrease between 2011 and 2022. This reduction was achieved through measures like stopping routine preventative use, limiting group treatments to situations when diseases are spreading and requiring susceptibility tests before prescribing certain antimicrobials. The newest Écoantibio plan (2023–2028) continues these efforts by aiming to further reduce use, particularly in companion animals, and focusing on rational use to maintain animal health (31). The Belgian Veterinary Surveillance on Antimicrobial Consumption National Report on Sales and Use of Antibacterial Veterinary Medicinal Products plays a key role in shaping Belgian policy on reducing the sale and use of antimicrobials in animals. The publication of these reports supports informed decision-making by providing authorities, representatives of the veterinary and agricultural sectors and other stakeholders with the data needed to set reduction targets. The goals for this policy were defined in the Vision 2020 and Vision 2024 programmes. The overarching goal of Belgium’s Vision 2024 was to reduce the use of antimicrobials in all animal species and by all veterinarians to a minimum. The next edition of this programme, Vision 2030, is currently being implemented in this country (1).

In the Polish context, policy efforts could be further strengthened by implementing targeted benchmarking systems for high-consumption sectors, particularly intensive poultry and pig production. Mandatory susceptibility testing of bacterial isolates for selected critically important antimicrobials, especially third- and fourth-generation cephalosporins and fluoroquinolones, could further support more evidence-based prescribing. Promoting vaccination programmes and farm-level biosecurity improvements in large-scale production systems may help reduce reliance on group treatments administered *via* drinking water. Finally, improving access to species-specific antimicrobial use data within the ESUAvet framework would enable more precise identification of high-consumption sectors and facilitate targeted interventions.

Because addressing antimicrobial resistance requires a coordinated effort from the human and animal health sectors worldwide, data are analysed and compared on combined human and animal antimicrobial consumption (AMC) from EU/EEA countries. The EU agencies deliver their findings in the joint inter-agency antimicrobial consumption and resistance analysis (JIACRA) reports (4). These reports analyse data from humans and food-producing animals from the agencies’ five EU-wide monitoring networks to facilitate better understanding of the occurrence of antimicrobial resistance across Europe and reveal any emerging trends, providing valuable insights for policymakers across the EU. The reports focus on specific combinations of antimicrobials and bacterial species considered important for public health. The JIACRA reports for the period 2014–2021 show the average total AMC in mg/kg of estimated farm animal biomass decreased by 44%, while it remained relatively stable in humans (4). Antimicrobial consumption values varied significantly across EU/EEA countries, with most, but not all, showing a decreasing trend. This demonstrates that measures implemented to reduce AMC in farm animals and humans were effective in many countries. However, these measures need to be strengthened to maintain the reduction and improve it if necessary. The results published in the JIACRA reports show that overall reductions in AMC can help reduce antimicrobial resistance. This also highlights the importance of actions that promote human and animal health, such as vaccinations and improved hygiene, thereby reducing the need for antimicrobials. Furthermore, data from 2019 to 2021 confirmed the association between the consumption of certain antimicrobial groups and the occurrence of antimicrobial resistance in bacteria isolated from both humans and food-producing animals. The fourth and latest JIACRA report notes that in food-producing animals, statistically significant reductions in the consumption of third- and fourth-generation cephalosporins, quinolones, polymyxins, aminopenicillins and tetracyclines occurred between 2014 and 2021 in at least a quarter of the countries analysed. During the same period, statistically significant trends indicating a reduction in human consumption of quinolones and aminopenicillins were observed in at least two-thirds of the countries analysed (4). These data underscore the need for continuous, integrated monitoring of AMC and AMR. These findings provide an important interpretative framework for the sales-based results presented in this study and further support the relevance of integrated One Health approaches in antimicrobial stewardship.

## Conclusion

This study provides a comprehensive analysis of sales of VMPs primarily for administration to food-producing animals in Poland in comparison with European trends over the period 2011–2023. The results reveal a clear divergence between national and EU-level patterns. While most European countries achieved substantial and sustained reductions in antimicrobial sales during this period, Poland recorded an overall long-term (between 2011 and 2023) increase of 19.5%, despite the implementation of EU regulations and national measures aimed at combatting AMR. However, this long-term increase masks a marked decline observed in recent years. Between 2019 and 2023, antimicrobial sales in Poland decreased by approximately 18.5%, suggesting a potential shift in the trajectory and the emerging effects of regulatory interventions and antimicrobial stewardship efforts.

The observed increase in antimicrobial sales in Poland over the analysed period is likely influenced by structural factors, including changes in animal population size, production intensity and species structure, particularly the dominant role of intensive poultry production. Although population-based indicators such as mg/PCU partially account for these differences, they do not allow for a precise assessment of antimicrobial use at the species or farm level. Further development of the ESUAvet system and access to species-specific usage data will therefore be essential for a more accurate evaluation of national AMC patterns.

At the European level, the sustained decline in antimicrobial sales confirms the effectiveness of long-term regulatory strategies, surveillance systems and awareness-raising initiatives promoting prudent and responsible use of antimicrobials in veterinary medicine. In contrast, the historically higher and more persistent sales levels observed in Poland underline the need to further strengthen national antimicrobial stewardship programmes, improve prescription control and enhance awareness among veterinarians and livestock producers. Structural shifts observed in Poland, including the growth in sales of oral liquid formulations and a reduction in sales of oral powders, indicate partial alignment with EU recommendations. However, the continued use of preparations in critically important antimicrobial categories underscores the necessity of ongoing monitoring and policy refinement.

To align with the EU target of a 50% reduction in veterinary antimicrobial sales by 2030, Poland should prioritise the expansion of antimicrobial stewardship initiatives, promote non-antibiotic disease prevention strategies such as vaccination and biosecurity and reinforce collaboration between veterinary, human and environmental health sectors within the One Health framework. Sustained and coordinated efforts in these areas will be essential to preserve the effectiveness of antimicrobials and to mitigate the growing public health threat posed by AMR.

Finally, the interpretation of these findings should take into account the inherent limitations of sales-based surveillance systems. Reported sales volumes may be influenced by factors such as cross-border trade in VMPs and national distribution structures, particularly in countries located at the external borders of the EU. For these reasons and others, sales data do not always directly reflect the actual use of antimicrobials in clinical practice and on farms. These limitations highlight the need for complementary data sources to support more precise assessments of antimicrobial use in veterinary practice.
